# Clinique Medicale, &c.; or, Select Cases Treated in the Charite Hospital

**Published:** 1835-10-01

**Authors:** 


### Clinique Medicale, ou Choix d'Observations recueillies a l'Hôpital de la Charité. Par *G. Andral*, &c. Deuxieme Edition, revue, corrigée, et augmentée par l'Auteur. Tom. V. Maladies de l'Encephale. Londres, Dulau, Soho Square.

[Third Article, continued from our last Number, p. 106.]

### Cerebral Hæmorrhage.

We shall now present our reader with a few of the more interesting cases of cerebral hæmorrhage.

Case 1.—*Sanguineous Clots scattered through the Substance of the Circumvolutions of the Brain—sudden Loss of Intelligence—Resolution of the Extremities—Death in the midst of a State of Coma,* 50 *Hours after the first Appearance of the Attack.*

A man, 49 years of age, was brought to the Maison de Santé in the following state:—State of coma, from which he could not be aroused—he seems as if plunged in a profound sleep; inspiratory movements succeed each other at long intervals; the four extremities, when raised, fall as inert masses; the severest pinching has not the slightest effect in making them move, nor is the expression of the countenance at all affected by it—lips not dragged; pulse 57. Such was his state in the morning, when we visited him. His friends told us that this man, having been for some time subject to some slight disturbance in his intellects, and being addicted to the use of ardent spirits, had complained of severe dizziness of the head the day before we saw him. These coming on at two o'clock, he suddenly lost the power of speaking, seeing, and hearing, and at four o'clock he fell into the state just described.—Such was the account we got. A large bleeding was prescribed, sinapisms to the legs, and diluent drinks. The following morning his state was the same, except that the inspiratory movements, which were so very slow the day before, were accelerated, without the frequency of the pulse being increased. Thirty leeches were applied to the neck, and the head was covered with a bladder full of ice. In the course of the day, the respiration became more and more embarrassed, and the patient died at four o'clock in the evening.

*Post Mortem.* Considerable injection of the vessels of the meninges. On each side, over the convexity of the hemispheres, the circumvolutions presented a sort of fluctuation in five or six points, each the size of a ten-sous piece. We had scarcely removed from one to two lines of the cerebral substance over these fluctuating points, when we found immediately beneath it some blood, slightly coagulated, contained in a small cavity, capable of containing a large hazel-nut. Around this cavity, the parietes of which were of a yellow colour, the cerebral tissue presented a bright red dotted appearance, but without any change of consistence. There were also from seven to eight small clots, seated in the circumvolutions of the upper surface of the right hemisphere, and nearly as many on the left. We found no other lesion in the encephalon. Walls of the heart were obviously hypertrophied.

*Remarks.* This case presents the very rare example of a case of hæmorrhage of the cerebral circumvolutions, without complication of any other effusion of blood into the remainder of the encephalon. The individual who was the subject of it, had nearly the same symptoms as those which are usually produced by a violent hemorrhage taking place in the substance of one of the hemispheres. Dizziness preceded the attack of apoplexy; the latter presented as a predominant phenomenon, the suspension of the senses, and of the faculty of speech; then there came on a state of coma, in which the patient died, fifty hours only after the appearance of the first symptoms. The respiration was accelerated only towards the termination, and the pulse continued free from frequency up to the last. The absence of hemiplegia is accounted for by the presence of the apoplectic clots (foyers) in both hemispheres. We shall not forget to remark that, around each of these clots, there existed, for the space of some lines, a bright red injection of the cerebral pulp. The nature of the effused blood attested the recent date of the hemorrhage.

Thus, simple compression of the most superficial part of some cerebral circumvolutions is sufficient to abolish intelligence, to suspend the exercise of the senses and of speech, and to produce a coma which proved rapidly fatal. We find the immoderate use of alcoholic liquors a predisposing cause of cerebral hemorrhage. The commencing alteration of the intellectual faculties observed before the attack is not accounted for by the state of the brain. The coincidence of hypertrophy of the heart, and of cerebral hemorrhage, should not be forgotten.

CASE 2.—*Cerebral Circumvolutions transformed into a sort of Erectile Tissue—small Sanguineous Effusions into this Tissue—Varicose Dilatation of the Veins of the Pia Mater—Perforation of one of them—Attack of Apoplexy. Death at the end of* 24 *Hours—Gangrene of the Lung.*

A woman, 50 years of age, entered the La Charité during the month of October, 1820, in a state of emaciation, the organic cause of which could with difficulty be appreciated. No cough—no dyspnœa—dull sound under the left clavicle on percussion—respiratory murmur more obscure there than in any other part—no sweats—pulse habitually a little frequent—tongue pale and moist—appetite gone. For three weeks, we saw this woman waste away more and more every day; at the end of this time, she was suddenly seized with attacks of vertigo, which were succeeded by loss of consciousness after two hours. At the morning visit, her intellectual and sensorial faculties appeared entirely suspended—the limbs, when raised, fell by their own weight—those of the left side insensible to pinching—the right not so much so—respiration stertorous—pulse hard, but free from frequency. Death the same day, towards evening.

*Post Mortem.—Cranium.* Veins ramifying over the net-work of the pia mater, covering the cerebral circumvolutions, were very much dilated at intervals—these were genuine varices; their parietes, soft and friable, were torn, and reduced to a kind of pulp by the least force. A layer of coagulated blood, at least six lines in thickness, covered the entire upper surface of the right hemisphere. After subjecting these parts to a stream of water, it was ascertained that one of the large varicose veins, which traversed the pia mater on the right, was perforated; it presented a large orifice, with irregular jagged edges, which was in some measure stopped by a small clot of blood. On the posterior lobule of the same right hemisphere, there were remarked four circumvolutions, which, at their surface, were changed into a bright red, areolated, fungus-like tissue, in the midst of which appeared three or four small cavities, each of which might admit a pea, and were filled with blood. In these parts the cerebral pulp presented not the least softening. *Thorax.* The summit of the right lung presented a portion, the size of an egg, black as ink, and changed into a liquid putrid substance, which exhaled an infectious odour. No communication as yet seemed to be established between this part, which was evidently gangrenous, and the bronchi.

*Remarks.* This is another instance of lesion of the encephalon limited to the circumvolutions. But their change differs in several respects from that presented in the first case. This alteration partly existed for a very long time before any cerebral symptom developed itself. The dilatation of the veins of the pia mater, and the softening of their wall, were certainly the effects of chronic disease. That remarkable development of the substance resembling erectile tissue on the surface of some circumvolutions was also a chronic affection. The production of this tissue depended, in all probability, on the dilatation of the capillary veins of the cerebral pulp, which were thus affected with the same kind of lesion, as the external veins in which they terminate. But what is very extraordinary, all this morbid process went on without any appreciable disturbance of the cerebral functions resulting from it. Then a period arrived, when there was a simultaneous laceration of one of the large external veins, and several of the small veins, the dilatation of which gave to some cerebral circumvolutions the appearance of erectile tissue. Thence resulted a double effusion of blood, the one being in the pia mater, the other in the pulp of the circumvolutions; and then only was it that the cerebral symptoms appeared. Violent vertigo was succeeded by a complete abolition of the senses and of intelligence; then coma supervened, in which the patient died. The loss of motion in one side of the body, and its preservation on the other, shewed that only one hemisphere was the seat of lesion; which is contrary to what occurred in the first case. We may remark here that there was no symptom from which we could have suspected the gangrene of the lung observed in this case.

CASE 3.—*Effusion of Blood into the Anterior Lobe of the Left Hemisphere of the Brain—Hemiplegia on the Right Side—Remarkable Embarrassment of Speech—Death on the 9th day.*

A labourer, 57 years of age, entered the La Charité in the following state:—intelligence very dull—extreme difficulty in pronouncing his words: he commences several phrases, without being able to finish any; after articulating with great difficulty some few words, there is nothing afterwards heard but an unmeaning stammering. When he is spoken to for any time, he laughs and cries alternately. He can give no account of what happened to him. Face red, eyes injected. Left commissure of lips drawn up. This side of the face alone is moveable; still he complains equally, whether the left or the right side of the face be pinched. Cannot by any effort put out his tongue. The two extremities of the left side are moved with ease; whilst those of the right side are deprived of all voluntary motion; the cutaneous sensibility does not appear to be diminished there. We find that four days previously this person fell down in the street deprived of consciousness, about 11 o'clock in the morning; that when brought home he did not recover it till the following day; and that since he has continued in the state described. During the four days following the tongue became dry and black, urine passed involuntarily, pulse frequent, extremities cold, respiration embarrassed, and he died nine days after the attack of apoplexy.

*Post mortem.—Cranium.* The portion of cerebral substance which terminates anteriorly the left hemisphere, is marked by a cavity which might contain a pullet's egg, and which is filled by a large clot of blood. This cavity commences an inch below the upper surface of the hemisphere, and a half an inch from its anterior extremity; posteriorly and inferiorly it is bounded by the portion of cerebral substance which forms the point of junction of the superior and anterior walls of the left lateral ventricle. Both this latter and the corpus striatum are perfectly intact. The parietes of the accidental cavity present on their internal surface a bright yellow colour which is continued two or three lines in depth. The cerebral substance nowhere softened. No other appreciable lesion in the brain. *Thorax.* Lungs infarcted, and *hepatized* also in several parts—heart large—hypertrophy of the left ventricle. *Abdomen.* Red softening of the gastric mucous membrane in its left half—slate-coloured tint of the pyloric portion. Numerous arborisations in the small intestine—spleen large and very soft.

*Remarks.* This case has been cited, as an instance of hemorrhage exactly limited to one of the anterior lobules of the hemispheres. According to the theory which attributes the direction of the movements of the lower extremities to the anterior part of the cerebral hemispheres, we should only have found here paralysis confined to the lower extremity of the right side, and yet the upper extremity of the right side was equally deprived of motion. The paralysis extended also to the right side of the face, and the motions of the tongue could no longer be performed. The articulation of the words was become very difficult—a circumstance which is found in accordance with the opinion put forth by Dr. Bouillaud with respect to the encephalic seat of speech. We content ourselves with noting here these different facts, proposing to ourselves, in our recapitulation, to add them to others for the purpose of resolving the important questions to which we have just referred. At the same time that the power of motion was abolished in one side of the body, sensibility was preserved intact on that side. With respect to intelligence, it was singularly dull; memory seen gone altogether. The commencement had been marked by a sudden loss of consciousness, and here again there was a coincidence between a cerebral hemorrhage, and a hypertrophy of the heart. It was not in consequence of this hemorrhage that the patient died. The perfectly sound state of the brain around the apoplectic clot was a good condition for the absorption of the effused blood. Death was the result of a twofold inflammation, that of the lungs, and also of the stomach, which particularly developed itself by the adynamic state into which the patient so suddenly fell. Such a mode of death is very rare in apoplectic subjects.

CASE 9.—*Effusion of Blood into the Corpus Striatum of the Right Side.*

—*Sudden Loss of Consciousness—Hemiplegia on the Left—Death the 15th day.*

A woman, 48 years old, addicted to wine, fell to the ground suddenly deprived of consciousness, on the 16th of March, 1823. A little after she was bled—at the end of two hours she came to herself—she entered the La Charité the same evening. On the next morning we found the two extremities of the left side completely deprived of motion and sensation. The right commissure of the lips was drawn upwards—intellect perfect—pulse hard, vibrating, and a little frequent. Blisters were ordered to the legs, and purgative mixture. On the following day a visible amendment. Sensibility restored in the paralysed side. The left lower extremity begins to perform some movements. The left upper extremity as much paralysed as on the preceding day. The 19th, she moves the leg and thigh of the left side with ease. Pulse not frequent. (A blister between the shoulders.) From this period to 1st April symptoms of gastro-intestinal irritation manifested themselves; tongue red and dry; great thirst; tension of the abdomen; diarrhœa. Delirium soon came on; the patient died in what is called the adynamic state. The paralysis of the lower extremity of the left side had been completely removed, not so of the upper.

*Post-mortem.—Cranium.* The only lesion presented by the encephalon was in the right corpus striatum. Towards the middle part of this substance, some lines beneath its upper surface, was found a small cavity filled with clots of blood. Around them the cerebral pulp was very soft for the space of three or four lines. *Thorax.* Hypertrophy of the walls of the left ventricle of the heart, with contraction of its cavity. *Abdomen.* Gastric mucous membrane very soft and red through the entire splenic portion. Intense redness, and as it were granular appearance of the inner surface of the ilium, through a great portion of its extent.

*Remarks.* It is rare to find hemorrhage so exactly limited to the corpus striatum, as in the above case. The commencement of the affection was similar to that of the generality of cerebral hemorrhages, whatever be their seat. The sanguineous effusion being inconsiderable, the patient soon recovered the use of her senses, and her intelligence continued quite perfect, which in this case may be referred to the seat of the hemorrhage having taken place far from the substance of the circumvolutions. At first the two extremities of the side opposite to that of the sanguineous effusion were equally paralysed, which already invalidates the opinion according to which isolated lesions of the corpus striatum should modify motion only in the inferior extremity. But this is not all; one of the paralysed limbs soon recovers the power of moving, and that is the lower extremity, that is to say, that limb which, according to the opinion just now mentioned, should alone have continued deprived of motion. Thus the more we advance, the more will these facts come to destroy, or at least to stagger assertions too hastily made. There was no appearance in this case of any curative process having been set up around the hemorrhagic cavity. The most alarming cerebral symptoms had however ceased, and it was under a complication of gastro-intestinal inflammation that the patient sank. She too had hypertrophy of the heart.

Having thus selected from our author's cases such as seemed most likely to engage the attention, we shall now proceed to the general results and inferences which he has drawn from them. He first considers the functional disturbances occasioned by cerebral hemorrhage, and then endeavours to ascertain how far the differences in these disturbances may be explained, either by the extent of the effusion, or the difference of its seat. And first he considers the—

*Lesions of Motion.* The most characteristic symptom of cerebral hemorrhage is paralysis, there being no instance known of hemorrhage into the cerebral parenchyma which was not accompanied by some affection of the faculty of motion. This paralysis appears at the moment the blood is effused—it presents great varieties with respect to its seat, varieties which pathological anatomy is very far from being able to explain. It may be divided into general paralysis, and that which is partial. The first exists, when the two sides of the body, whether entirely, or in some of their parts, are at once deprived of motion. This general paralysis may occur in the three following cases:—

1st. In the case of simultaneous or successive hemorrhage into the two hemispheres. 2d. In the case of considerable hemorrhage into only one hemisphere, with destruction of the parietes of the corresponding lateral ventricle, escape of blood into this ventricle, and from this into the other cerebral cavities, either through the natural orifices of communication, or through the lacerated septum. 3dly. In the case of hemorrhage into only one hemisphere, without effusion of blood into the ventricles, but considerable enough to have broken down the greatest part of the substance of this hemisphere. In the case of general paralysis, the four extremities fall as inert masses, and we may always observe loss of consciousness and profound coma inseparable from it—this species of paralysis however indicates with less certainty the occurrence of cerebral hemorrhage, than that of one half the body; the latter form being one of the surest signs of hemorrhage into the brain. The paralysis consequent on cerebral hemorrhage, is that of the two extremities of the side opposite to that where the effusion takes place. When the two extremities are simultaneously paralysed, they may be so in an equal degree, which is very rare; for it is in the upper extremity that the loss of motion is in general most complete. The moment when the hemiplegia occurs coincides in many cases with complete loss of consciousness, and then the individual so attacked may fall in any direction. But when this loss of consiousness does not take place, the patient suddenly feels the lower extremity which has been just paralysed suddenly withdrawn from under him, and he falls on the side of the hemiplegia, though he still preserves his consciousness. Hemorrhage of the cerebral hemispheres may also merely produce paralysis of a single limb, sometimes of the upper, and sometimes of the lower extremity. Recently some facts have been published with the intent of proving that paralysis of the upper extremities depends on a lesion limited to the optic thalami, or to the nervous mass on their level, and behind them, and that paralysis of the lower extremities depends on lesions of the corpora striata or of the nervous mass, on a level with and anterior to them. M. Andral endeavoured to ascertain the truth of this theory, and to that end he selected 75 cases, in which the hemorrhage was so circumscribed as to qualify them for assisting in the solution of the question. Out of these 75 he found 40 in whom the two extremities of one side were simultaneously paralysed. Of these 40 there were 21 in whom the seat of lesion was in the anterior lobe, or corpus striatum, and 19 in whom the lesion was seated in the posterior lobe, or optic thalamus. Of these same 75 cases we found 23 in whom the paralysis was confined to the upper extremity, of whom eleven had lesion of the corpus striatum or anterior lobe, 10 with lesion of the optic thalamus or posterior lobe, 2 with lesion of the middle lobe.

Again, of these same twenty-five cases, we found twelve in which the paralysis was confined to the upper extremity, in ten of which the seat of the lesion was in the corpus striatum or anterior lobe, whilst in two the lesion was in the optic thalamus or posterior lobe.

From these facts we are led to the conclusion, that in the present state of science, we cannot yet assign in the brain a distinct seat for the motions of the upper and lower extremities. No doubt such distinct seat exists, since each of these extremities may be paralysed separately, but we do not yet know it.

At the same time that the extremities of one side of the body are paralysed, other parts also may be similarly affected, but in different degrees; these parts are commonly the following; the globes of the eyes—the eyelids—the different parts of the face—the lips—the tongue—the neck—the larynx—the pharynx and œsophagus—the bladder—and the rectum.

Of all these parts there are none whose paralysis is as common as that of the extremities, nor does it present itself with equal frequency in the other parts. Thus the different parts of the face, and the tongue are more frequently the seat of it, than the other parts above-mentioned.

Paralysis of the muscles which move the eyes is denoted by the constant deviation of the latter in some one direction; strabismus is then generally observed; but this phenomenon itself is very rare, and it is scarcely ever noticed in the numerous cases published on cerebral hemorrhage. We have ourselves met it but seldom. In order to its production it is necessary that the muscles antagonizing those which carry the eye downwards and inwards should be paralysed.

The muscles forming the parietes of the cheeks lose the power of contraction much more frequently than the preceding, in cases of cerebral hemorrhage. The buccinator is the muscle whose paralysis is most perceptible. Every time the patient expires, one of the cheeks is observed to be passively distended, and also at the same time one half of the lips corresponding to it; and when subsequently the patient wishes to masticate, the food introduced into the mouth and placed on the side of the paralysed buccinator can be no longer acted on by it, and becomes collected between the cheek and the teeth, until removed by mechanical force.

In all the cases we have met, and in all those published with sufficient detail, the paralysis of the buccinator muscle has taken place on the same side as that of the extremities.

The passive distention of one cheek we generally found to take place only in very severe cases, and where there was at the same time loss of consciousness.

The muscles which move the lips often retain all their power of motion, so that the commissures present no deviation whatever. But at other times these muscles are paralysed, and in consequence of their antagonizing power being destroyed, the commissure is drawn to the opposite side and outwards, and at the same time it inclines sometimes upwards and sometimes downwards. In the great majority of cases the deviation of the commissure is towards the side opposite the hemiplegia; it is consequently on the same side as the paralysis of the limbs, that the paralysis of the muscles takes place by which the lips are moved. Oftentimes when no motion occurs, and as long as the mouth remains closed, there appears no deviation; but this becomes perceptible, when the patient speaks or smiles. The degree of deviation of the mouth is not always in the direct ratio of the degree of hemiplegia. We have seen it very marked in cases where the paralysis of the limbs was but slight; whilst we have found it absent, where the hemiplegia was complete—

The tongue with respect to its movements presents very different states in individuals attacked with cerebral hemorrhage. In the first place its movements may remain perfectly free. Persons, after having continued several minutes unable to move it, appear suddenly to recover this power: they protrude it abruptly from the mouth after a great effort; but there are some movements which they cannot give the tongue until after considerable intervals—some cannot protrude the tongue—some are unable to articulate—others who are able to protrude it, cannot put it straight from the mouth. According to M. Andral's experience the apex of the tongue always deviates to the paralysed sides. A consideration of the anatomy of the muscle by which the tongue is protruded will show that such must be the case. Paralysis of the muscles of the neck is but very rarely observed in cerebral hemorrhage. The head then is observed to incline to the paralysed side, whilst the face is turned to the opposite side. Paralysis of the respiratory muscles is only observed in that species of apoplexy, called from its severity and the rapidity of its effects, *apoplexia fulminans*. Aphonia is sometimes observed, owing to paralysis of the internal muscles of the larynx. Paralysis of the muscles of the pharynx and œsophagus is observed only in the most serious cases, and is always a fatal symptom. Paralysis of the bladder is by no means so common as has been asserted. We shall return to the subject, when we come to consider the sensibility of the different parts as affected by cerebral hemorrhage, to which, as being peculiarly interesting in a practical point of view, we shall now direct the reader's attention.

### CHAP. II.—LESIONS OF SENSIBILITY.

These lesions are much more constant in cases of cerebral hemorrhage than those of motility; and, up to the present time, it has been impossible to detect, in the nature or in the seat of the alterations of the brain, the cause which sometimes suffers the sensibility to be intact, and sometimes produces its more or less complete abolition.

We shall now consider these lesions of sensibility: 1mo, in the skin; 2do, at the surface of the different mucous membranes capable of being touched; 3tio, in the organs of sight, of hearing, of taste, and of smell; 4to, in the encephalon itself.

1. *Lesions of the Cutaneous Sensibility.* These must be considered at two periods—both before the hemorrhage has taken place, and after its occurrence. Before the period when the hemorrhages come on, many persons experience nothing particular towards the cutaneous periphery, but with others it is not so. The pulp of the fingers becomes the seat of divers sensations: several complain of having in their feet a singular feeling of cold, a sort of numbness, similar to what is felt when the hand is plunged into the frozen water—others complain of pricking sensations, or annoying formication towards the extremity of the fingers; others, again, fancy that there is a piece of cloth interposed between the skin of their fingers and the body, which they would touch; so much blunted is their sensibility. These different sensations may be confined to the hands, they may extend to the feet—they may even manifest themselves at other points, either of the extremities or of the face or trunk. We have seen the case of a man, who, before being struck with apoplexy, experienced, from time to time, complete loss of sensation in some isolated points of the skin of the thorax. Each of these points, which were to the number of five or six, might be about the size of a five-franc piece. There the skin might be pinched ever so severely, without the patient seeming to feel the slightest pain; outside these points the sensibility was intact, and it soon re-appeared in all its integrity. These partial abolitions of the sensibility were not constant; there were some days when the sensibility was not diminished in any part—then, suddenly, it disappeared from the parts just now mentioned. Another patient after having left the La Pitié, where he had been treated for an intense erysipelas, principally seated in the left side of the face, cranium, neck, and back, entered again at the end of two months, with total loss of the sensibility of the different parts of the skin, where the erysipelas had been. Thus, the skin of the face of the left side, that of the scalp of the same side, and also that of the neck, from the median line to the level of the top of the shoulders, had lost all sensation. On this same side, hearing, sight, smell, and taste were also nearly destroyed; the motility of the parts affected had not, however, undergone any change. This patient had, for the last six weeks, experienced almost constant dizziness, and it was nearly since that same period, that he commenced to lose the sensibility in all the portions of the skin previously affected with erysipelas. Was there not, in this case, a specific affection of the fifth pair of nerves?

The perversions of cutaneous sensibility, preceding the apoplectic attack, may develop themselves always in the same point, or seize on different parts one after the other; they may manifest themselves on both sides of the body at once, or confine themselves merely to one, and, in the latter case, the side which they affect will, in general, be that which, at a subsequent period, will become paralysed.

Nothing is more uncertain than the time intervening between the lesion of the sensibility, and the apoplectic attack. In several cases, we have seen the sensibility modified only a few days before the appearance of the symptoms of cerebral hemorrhage: in others numbness, formications of the extremities, manifested themselves some years before the occurrence of the hemorrhage.

Let us now trace the modifications which the cutaneous sensibility may undergo, after the cerebral hemorrhage has appeared.

The abolition of sensibility does not always accompany loss of motion.

When it does take place, it is generally seated in the parts whose power of motion has been modified. We have seen some cases, however, in which it was not so. In a man, for instance, who entered the La Pitié in the year 1831, we made the following observations:

This person, 60 years of age, after having for a long time experienced a pain of the head, seated principally in the right anterior lateral part of the cranium, suddenly lost the faculty of seeing on the same side, where the headache existed; and, at the same time, the pupil of the right eye was contracted in an extraordinary manner. For about six weeks, the right eye remained thus deprived of sight, without any other phenomenon appearing. But, at the end of this time, the right side of the face lost all sensibility, without its motive powers being in any way changed. From fifteen to twent[illegible] days, the sensibility remained thus lost on the right side of the face, then the upper extremity of the left side became evidently weaker than the other, without the cutaneous sensibility in this extremity being changed, and, nearly at the same period, the muscles of the right side of the face began to become paralysed, and the mouth was slightly drawn towards the opposite side. There was not, in this case, the slightest loss of consciousness.

We shall not here discuss what the lesion was, which, in the individual whose case we have just now mentioned, produced this double modification of sensation and motion; we mentioned the case, merely to point out the extraordinary circumstance of loss of sensation on the right, and of motion on the left. In this case, again, there is another peculiarity—it is this, that the paralysis of motion came on in the left side of the face, that is, in the side opposite that of the paralysed limb. Now this circumstance establishes a striking exception to the law which we previously laid down.

According to the time which elapses from the apoplectic attack, the cutaneous sensibility re-appears, and most usually it is found almost completely re-established, at a time when the paralysis of motion still remains undiminished. However, the fingers often continue benumbed and cold; but that may depend, in a great measure, on the privation of motion producing a degree of languor in the capillary circulation.

2. *Lesions of the Sensibility of the Mucous Membranes.* In those cases where the sight is lost, the eyelids are made to approximate by touching the conjunctiva with the finger. There are, however, some cases in which this membrane becomes so insensible, that the end of the finger may be passed over the entire anterior surface of the globe of the eye, without the eyelids approximating, or without the patient's evincing the slightest sign of pain, and that at a time when, in other parts, the sensibility is still sufficiently acute. One of our cases furnished us with a very remarkable instance of that kind. This insensibility is similar to that which may be produced in animals by dividing the fifth pair of nerves.

On introducing a feather into each of the nostrils, we ascertained, in some apoplectic patients, a notable diminution of sensation on one of the halves of the mucous membrane of the nasal fossæ. Some have told us that, on introducing their food into each side of the mouth alternately, there was one side in which the contact of the food with the mucous membrane of the mouth was felt much less distinctly than in the other. We particularly ascertained this diminution of sensibility of one of the sides of the mouth, and one of the nostrils, in an individual, the corresponding side of all whose face was likewise deprived of sensibility, whilst the power of motion remained intact; in no other part was it altered; sight and hearing were also much weaker on this same side than on the opposite side.

These different modifications of sensibility seem to indicate that there are cases where hemorrhage of the cerebral hemispheres, whatever be its seat, exercises an influence on the fifth pair of nerves.

3. *Lesions of the Functions of the Organs of the Senses.* In a considerable number of cases, vision is not modified. When it is disturbed it may be so before the hemorrhage takes place, at the very time when it has occurred, or after its occurrence.

Before the hemorrhage, several persons experience on the part of vision, strange sensations, real hallucinations. With some all objects appear to be coloured red; others fancy that a red line borders all bodies; a sensation similar to what is experienced, when the eyes have been for some time exposed to the impression of a strong light. There are some who cannot look attentively at an object without seeing it dotted with red or black points; others have a constant mist interposed between their sight and the object they are looking at. Some are tormented by the appearance of this which seem to them constantly dancing before their eyes.

It must not however be supposed that these different hallucinations of vision lead necessarily to cerebral hemorrhage. We have had an opportunity of observing a person, who, for several years, was constantly tormented by the imaginary sight of small bodies of different forms and colors dancing before his eyes: if he would look steadily at an object, he saw it dotted with a number of black points; this hallucination which was permanent, prevented his being able to read or write; he had neither dizziness, vertigo, nor headache; the conjunctivæ were habitually red, and he could not endure without considerable suffering a more than ordinary strong light.

Diplopia has been occasionally observed a certain time before the supervention of an attack of apoplexy.

Other persons have been suddenly struck with blindness, and the loss of sight was with them the principal precursor of cerebral hemorrhage. We have seen a locksmith, who after having experienced considerable dizziness of the head for eight days, suddenly lost his sight. After having remained blind for fifteen days, he suddenly fell down deprived of consciousness, and paralysed on the right side; consciousness soon returned; the hemiplegia continued; but, what was very remarkable, some time after the attack, he began to recover his sight, which however continued very weak with him. We saw another individual in whom, during the month preceding the attack, the sight was completely lost three different times; he suddenly became blind; the blindness continued from forty-eight to sixty hours; he recovered his sight.

In a word, individuals have been observed in whom for some time before the attack, their vision acquired unusual sharpness.

Such are the principal phenomena which manifest themselves as connected with sight, a longer or shorter time before the hemorrhage supervenes. Their existence proves indisputably that before the blood is effused, there is already going on in the brain a morbid process, either continued, or intermittent, the nature of which it would be a matter of great importance precisely to determine.

Once the hemorrhage has come on, the sight may remain unaffected; but it may also be lost. Sometimes it is lost on both sides; that takes place in violent apoplexy, when the hemorrhage is very extensive. Sometimes, on the contrary, the power of vision disappears only on one side. But here two different cases have been observed: in the one case the sight is lost on the side where the paralysis of the limbs exists: in the other case the patient does not see with the eye of the side opposite the paralysed side of the body.

We have investigated how far the hemorrhage occupied a particular seat in those cases, where, after its occurrence, the sight continued affected, and this seat we have never been able to discover. We might cite cases here either from our own practice, or which have been recorded by other writers, in which we might find different alterations of vision, though the most different parts of the hemispheres were the seat of the hemorrhage. We do not admit then with M. Serres, that sight is lost only when the hemorrhage has its seat in the optic thalami, on the level of the commissure. We shall see as we go on, that lesions of the cerebellum are also accompanied with different disturbances of vision, and in particular with amaurosis. In the face of so many facts, which show us constantly, in the alterations of the brain, the most different seats, to explain the disturbance of one and the same function, shall we deny that certain parts of the brain are particularly destined for the performance of certain acts? We would have no right to do so; for it is probable that certain points of the brain have such a relation among them, that the lesion of such a one among them will re-act in particular on such another; and this will probably be the secondary alteration of the latter, inappreciable by the scalpel, which is to produce the peculiar functional disturbance.

The sense of hearing may present, before, during, and after the cerebral hemorrhage, the same modifications as the sense of vision. Before the hemorrhage there are some who are annoyed with buzzing in the ears, continual or intermittent tingling. Several fancy they hear the strangest noises. These hallucinations are however far from being the constant prelude to an attack of apoplexy; they may be connected with mere perversions of sensibility, and have nothing whatever to do with cerebral congestion.

We have no particular observations to make on the modifications produced in the senses of taste and smell by hemorrhage of the cerebral hemispheres.

4. *Lesions of Sensibility seated in the Encephalon itself.*—Pain of head more or less intense, dizziness, vertigo, often precede cerebral hemorrhage. There are some persons, who, for several months, are constantly affected with the signs of cerebral congestion; one day it becomes more violent, and the hemorrhage takes place. We cannot understand how persons can deny such a precursor, and assert, that it takes place only in cases of softening. We acknowledge besides that it is often completely wanting, and that individuals may be suddenly struck with cerebral hemorrhage, without ever having before presented the least symptom as referrible to the brain, without having ever complained either of headach, dizziness, &c.

After the hemorrhage, there is no additional phenomenon observed, we merely see the same symptoms continue in a great number of cases (such as vertigo, etc.) as had been the precursors of the disease.

### Chap. III.—Lesions of Intelligence.

In the same manner as the lesions of motion and sensation, these also must be considered, before the hemorrhage has taken place, and after it has occurred.

Several persons preserve all the clearness and strength of their intelligence, up to the moment when they are struck with apoplexy. In others there are observed, a shorter or longer time before this period, some changes in the intellectual faculties; sometimes they are as it were benumbed; sometimes, on the contrary, they manifest an extraordinary excitement. Some patients lose their memory; there are moments when they know neither where they are, nor what they do, nor what they say. We here give some instances of these aberrations of intelligence, which we have had an opportunity of observing.

A woman, whose reason had been up to that period perfectly sound, gave herself up all at once, without any obvious cause, to violent fits of passion; she became frantic, and was conveyed to the La Charité, in a state resembling mania; on the very evening of her entering the hospital, she was struck with apoplexy, of which she died in less than thirty hours. On opening the body we found in one of the cerebral hemispheres an enormous effusion of blood.

A man, about 50 years of age, forgets his own name: he is from time to time convinced that he is dead; he no longer recognizes his own immediate relatives; he remains fifteen days in this state; then he is struck with apoplexy; the post-mortem examination again shows in this case an effusion of blood within the hemispheres; no other lesion was discovered.

Another man becomes incapable of attending to any occupation: he remains constantly seated, and his eyes as it were weighed down with sleep; we could with difficulty elicit from him some few answers: this state terminated by an attack of apoplexy.

Several similar cases have been seen by practitioners; they clearly prove that before the time the hemorrage takes place, there may be already in the brain a morbid state, which is the precursor of it, and which may manifest itself by divers disturbances of motion, sensation, or intelligence.

Other persons experience, at several different times, sudden losses of consciousness; they fall suddenly into a profound coma, and it is supposed that they are under the influence of cerebral hemorrhage. But this coma soon disappears, and they are restored to perfect health, until a new coup de sang returns. At last a time comes when, instead of a simple congestion by which all these phenomena might be explained, there comes on a real hemorrhage, the effects of which are no longer transient, as those of the congestion which preceded it.

Even at the time the hæmorrhage takes place, three cases may present themselves with respect to the modifications which the intelligence undergoes.

In the first case it is perfectly intact, and the serious alteration which the power of motion then suddenly undergoes, brings no disturbance on the exercise of the intellectual faculties.

In a second case, the intelligence becomes more or less dull, at the same time that the limbs are paralysed. The patient fall into a stupor; others form incoherent resolves, or utter some unintelligible words; however they are still conscious of the external world, and they are still able to hold relation with it.

In a third case, on the contrary, the loss of consciousness is complete. The patients are plunged into a state of coma from which the most energetic excitements cannot arouse them. Only sometimes, after being spoken to with a very loud voice for the purpose of awakening them, they open their eyes slowly, and stare for some seconds on the person who is watching them; but they soon relapse into their lethargic sleep.

These differences in the state of the intelligence at the time the attack of apoplexy takes place, depend principally on the greater or less extent of the effusion. With respect to the seat of the latter, it has not appeared to us to exercise any great influence on the intellectual faculties. Not only have we seen the loss of consciousness coincide with hemorrhage in all possible points of the cerebral hemispheres, but we have even found it in cases where the hemorrhage had its seat outside the hemispheres, in the cerebellum for example, or in the pons varolii. Dr. Fabre has cited the very interesting case of an old man, who died of an attack of apoplexy accompanied with complete loss of consciousness, in whom the nervous centres presented no other lesion than an effusion of blood into the substance of the left anterior pyramid; a very striking example no doubt of the wonderful connexion, which holds together, and brings into unity of action all the parts of the nervous system.

After the effusion of blood has taken place, the coma may remain, the patient does not recover consciousness, and, in this case, death soon arrives. In the most favorable cases, and which are far from being rare, the state of coma disappears; but once the individual has come to himself, the intelligence does not always present the same condition. In a very small number of cases, it is perfectly re-established; most frequently it remains enfeebled—the patient retains sufficient reason to be able to attend to the concerns of common life; but he has become incapable of deep or profound reflection—he can no longer join, without distress, in a conversation of any length, or of a serious nature, and it is necessary to debar him from it, otherwise his state may be made worse.

Instead of this simple weakness, the intelligence may present a more serious alteration. Thus, we see a great number of apoplectic patients fall into a real state of childishness, or of senile dotage; they shed tears with extraordinary facility. Others are seized, from time to time, with a delirium, which resembles that so often induced by acute inflammation of the meninges, and, in fact, it may be supposed that, in such cases, it is caused by the occurrence of an irritation of the arachnoid which covers the affected hemisphere. Madness, in a word, has been seen to declare itself after a cerebral hemorrhage.

There is a phenomenon observed rather frequently, after an effusion of blood into the brain, that is, loss of speech. It may exist with a perfect integrity of intelligence. Sometimes this accidental dumbness soon disappears—sometimes the speech is not recovered till after the expiration of a considerable time; sometimes, in a word, it continues for ever lost.

M. Professor Bouillaud published some years since a paper, containing some curious facts, for which he thought he might conclude, that the formation of speech has for its instrument the anterior extremity of each hemisphere, he having found this part the seat of lesion every time that, during life, speech itself had been lost. What our researches on this subject have led us to conclude, is as follows:—

Out of thirty-seven cases observed by ourselves or by others, relative to hemorrhages or other lesions, in which the morbid change in one of the anterior lobes, or in both, speech was abolished twenty-one times, and retained sixteen times.

On the other hand, we have collected fourteen cases, where the speech was abolished without any alteration in the anterior lobes. Of these fourteen cases, seven were connected with diseases of the middle lobes, and seven with diseases of the posterior lobes.

The loss of speech is not, then, the necessary result of the lesion of the anterior lobes; and, besides, it may take place in cases where anatomy does not shew any alteration in these lobes. M. Lallemand* has cited a case, in which there was found no other alteration than a softening of the white substance of the left lobe of the cerebellum; in this case, however, to which we shall take occasion to return for another purpose, the faculty of speech was completely lost. In M. Ollivier's work on the spinal cord,† you will find the case of an individual, in whom occurred the phenomenon of loss of speech, at first partial, and then complete: in this case, it was in the pons that the alteration existed; it was found softend at its lower surface, to an extent equal, at least, to the size of a filbert.

### Chap. IV.—Lesions of the Functions of the Organs of Nutritive Life.

Among these functions, there is but one which is specially affected by cerebral hemorrhage; and, again, the latter must be rather considerable, or, which comes to the same thing, it must find the individual so predisposed, that a slight effusion will produce in the brain a greater disturbance than would seem to be compatible with the intensity of the lesion. Then the respiration presents a particular character, which is designated by the term *stertorous*. Must we admit, with M. Serres, that this function is particularly affected, in the case in which the hemorrhage is seated in the optic thalamus and in its radiations?‡

The stertor of the respiration is, in general, a very fatal sign, and individuals who present it in a marked manner seldom escape a speedy death. To

---

* Letter ii. p. 134. † Tom. ii. p. 614.

‡ *Anatomie Comparée du Cerveau,* tom. ii.

account for it, there is found on the dead body considerable infarction of the lungs, and a great quantity of frothy mucus in the bronchi. It is certainly in consequence of the embarrassment of the respiration, that persons struck with cerebral hemorrhage die, in the case where the attack is severe, and where they die promptly.

The circulation presents various disturbances ; the heart frequently beats with strength, but this strength is rather in reference to its preceding state than to the cerebral disease itself. The pulse is variable—it is, however, more frequently slow than otherwise.

The digestive functions present no particular disturbance, except frequently obstinate constipation, which is not always overcome by drastic purgatives. It should be remarked, however, that the absence of alvine evacuations in such cases, as M. Andral observes, is far from indicating an *insensibility* of the mucous membrane to the action of irritating substances, brought in contact with it, inasmuch as a bright red injection has been frequently found on the internal surface of the intestine, arising, no doubt, from the irritation of the purgatives. There is an ambiguity in the term insensibility, which might be apt to lead the younger portion of our readers astray. M. Andral obviously alludes to the absence of that species of sensibility called *organic sensibility*. We remember that Lallemand, in his work on Diseases of the Spinal Cord, says that the sensibility of the urinary bladder is diminished—the patient makes no effort to expel the urine, " because he does not *perceive* the impression made by it on the mucous membrane ;" here Lallemand evidently means the *animal sensibility*. The same reason for the inflammation of the bladder, in the one instance, will hold good for the inflammation of the intestinal mucous membrane in the other. Another instance, in addition to that alluded to at the commencement of this present article, of the importance of precision and clearness in the use of terms, more particularly in philosophical discussions.

We have now selected, from the volume before us, those parts which have a practical bearing, and which are of daily, nay, hourly application, and, consequently, of primary importance to the practical physician. With respect to that bone of contention, which has set our French neighbours so often at loggerheads with each other, we mean " ramollissement" of the brain, some of them maintaining that it is inflammatory in its nature—others, that it is a morbid change, *sui generis*, we regret to say that, in the present state of science, little of practical utility can be said about it. We may, perhaps, return to it at some future period. Regarding the merits of this volume, we can say no more than we said in our last number: we arise from the analysis of it very much gratified, and we shall have no hesitation in saying, very much instructed. It is, in fact, a book which should be carefully studied by every medical practitioner.

